# The Genome-Level Survey of the WOX Gene Family in *Melastoma dodecandrum* Lour.

**DOI:** 10.3390/ijms242417349

**Published:** 2023-12-11

**Authors:** Ruiyue Zheng, Yukun Peng, Jiemin Chen, Xuanyi Zhu, Kai Xie, Sagheer Ahmad, Kai Zhao, Donghui Peng, Zhong-Jian Liu, Yuzhen Zhou

**Affiliations:** 1 Ornamental Plant Germplasm Resources Innovation & Engineering Application Research Center, Key Laboratory of National Forestry and Grassland Administration for Orchid Conservation and Utilization, College of Landscape Architecture and Art, Fujian Agriculture and Forestry University, Fuzhou 350002, China; fafuruiyue@163.com (R.Z.); pyk20001022@163.com (Y.P.); c2549539102@163.com (J.C.); zxy316377506@163.com (X.Z.); xiekai_0526@163.com (K.X.); sagheerhortii@gmail.com (S.A.); fjpdh@126.com (D.P.); 2 College of Life Sciences, Fujian Normal University, Fuzhou 350117, China; zhaokai@fjnu.edu.cn

**Keywords:** WOX transcription factors, physicochemical properties, expression analysis, plant growth

## Abstract

Though conserved in higher plants, the WOX transcription factors play crucial roles in plant growth and development of *Melastoma dodecandrum* Lour., which shows pioneer position in land ecosystem formation and produces nutritional fruits. Identifying the *WOX* family genes in *M. dodecandrum* is imperative for elucidating its growth and development mechanisms. However, the *WOX* genes in *M. dodecandrum* have not yet been characterized. In this study, by identification 22 *WOX* genes in *M. dodecandrum* based on current genome data, we classified family genes into three clades and nine types with homeodomains. We highlighted gene duplications of *MedWOX4*, which offered evidences of whole-genome duplication events. Promoter analysis illustrated that cis-regulatory elements related to light and stress responses and plant growth were enriched. Expression pattern and RT-qPCR results demonstrated that the majority of *WOX* genes exhibited expression in the stem. *MedWOX13s* displayed highest expression across various tissues. *MedWOX4s* displayed a specific expression in the stem. Collectively, our study provided foundations for elucidating *WOX* gene functions and further molecular design breeding in *M. dodecandrum*.

## 1. Introduction

WUSCHEL related homeobox (WOX) Transcription Factors (TFs) belong to the homeodomain superfamily of transcription factors. They contain a homeodomain that folds into a DNA-binding domain formed by 60 to 66 amino acid residues [1]. In *Arabidopsis thaliana* L., 15 *WOX* genes that participate in regulating early embryonic development have been identified. Phylogenetic analysis has categorized *WOX* genes into three evolutionary clades: ancient, intermediate and WUS [1,2,3]. However, plants with different taxonomic positions had different WOX branches. *Ostreococcus tauri* C. Courties & M.-J. contains only ancient clade, and this clade is found to be expanded in mosses [4]. In fern plants, WOX intermediate clade related genes were found to exist, such as in *Ceratopteris richardii* Brongn. [5] Whereas in higher seed plants, WUS clade WOX proteins were found to be present [6].

*Melastoma dodecandrum* Lour. is a creeping shrub widely distributed across southern China [7,8,9]. A pioneer species is a type of plant, fungus, or organism that is among the first to colonize or inhabit a newly formed or disturbed habitat [10]. As a pioneer plant, *M. dodecandrum* possesses typical advantages of pioneer species [11]. The stems of *M. dodecandrum* have numerous adventitious roots, which can help it adapt more easily to new habitats. A pioneer plant also can profoundly influence the growth environment and ecosystem [12,13]. Additionally, pioneer plants play important roles in plant community succession and ecotones, with many pioneer herbaceous plants being major agricultural weeds [14]. *WOX* genes can regulate shoot apical meristem formation and promote differentiation or maintenance of the vascular procambium [15,16]. Therefore, *WOX* genes may be related to the growth of plant stems. The presence of adventitious roots in *M. dodecandrum*’s stems enables them to anchor to the ground, leading to a creeping growth habit. Investigating *WOX* genes in *M. dodecandrum* is crucial for unraveling the mechanisms behind plant creeping growth and the emergence of pioneer plants.

Previous studies have demonstrated that *WOX* genes play an important role in plant growth and development. In *Rosa canina* L., *RcWOX1* play a pivotal role in auxin induced rhizoid formation [17]. *WOX5* interacts with *WOX1* and *WOX3* to control leaf shape in *A. thaliana* [18]. *WOX6* and *WOX11* regulate *Oryza sativa* L. tillering angle through auxin [19]. *WOX7* has been reported to regulate lateral root development and *WOX2* and *WOX8* involve early embryonic development in *A. thaliana* [20,21]. *NsWOX9* in *Nicotiana sylvestris* Speg. interacts with *LAMINA1* to regulate cytokinin levels, thereby regulating cell proliferation and differentiation [22]. *OsWOX10* involves the timely initiation and growth of rice roots [23]. *WOX11* played a mediating role in *SLG2*, allowing *SLG2* to specifically regulate grain width [24]. *WOX11/12* enhance salt tolerance in poplar tree by activating the *PagCYP736A12* gene [25] And the *WOX14* stimulates vascular cell differentiation and lignification in *A. thaliana* stems [26]. Moreover, The *WUS/WOX* genes could interact with *ICDH*, influencing plant stem cell maintenance in response to nutrient deficiency. It also could interact with *FINS1* to respond to fructose signaling [27,28]. *WUS* affects the development of plant embryos, bud meristem and reproductive organs [29].

*WOX4* and *WOX13* are key members of the *WOX* gene family that play vital roles in regulating plant growth. In *Liriodendron* hybrids, *LCWOX4* expression restricts to the vascular tissues of cotyledon embryos [30]. Silencing of *GhWOX4* retards the growth and development of *Gossypium hirsutum* L. [31,32]. WOX13 regulates plant stem cell growth, initiates callus formation, facilitates organ reconnection and negatively regulates shoot apical meristem formation from callus in *A. thaliana* [16,33,34]. The number of WOX genes varies across plant species. For example, 18 *WOX* genes are identified in *Helianthus annuus* L. [35], 14 in *Triticum aestivum* L. [36] and 9–14 members in Rosaceae species [37]. Additionally, the WOX gene family of many plants was discussed, such as *Gossypium*, Rosaceae species, *Pinus sylvestris* L. and *Raphanus sativus* L. [38,39,40,41] Although the roles of WOXs have been well-studied in a lot of plants, little is known about their function in pioneer plant. Therefore, we systematically identified and analyzed the characteristics of *WOX* genes of *M. dodecandrum*, including gene structures, conserved domains, phylogenetic tree, homologous and repetitive genes, cis-regulatory elements and expression patterns. We hope our research findings can provide novel insights into the study of plant creeping growth and pioneer plants.

## 2. Results

### 2.1. WOX Gene Identification and Protein Features in M. dodecandrum

**Figure 1:**
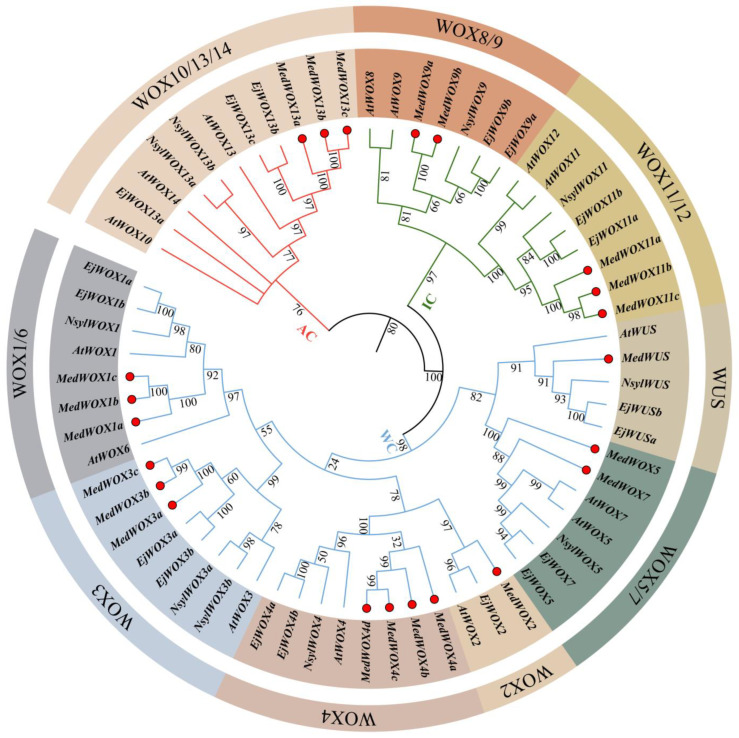
Phylogenetic tree of the WOX proteins from *Melastoma dodecandrum*, *Arabidopsis thaliana*, *Eriobotrya japonica* and *Nicotiana sylvestris*. The phylogenetic tree was constructed with the Maximum Likelihood (ML) method by PhyloSuite software and was divided into three clades and 9 categories.

After PFAM(PF00046) searching and BLAST analysis in TBtools, a total of 22 MedWOX members were identified, which were classified according to the phylogenetic relationships with their counterparts of *A. thaliana*, *Eriobotrya japonica* L. and *N. sylvestris* (Figure 1 and Table S5). We divided 22 MedWOX proteins into 9 categories, including WOX1, WOX2, WOX3, WOX4, WOX5, WOX9, WOX11, WOX13 and WUS.

**Figure 2:**
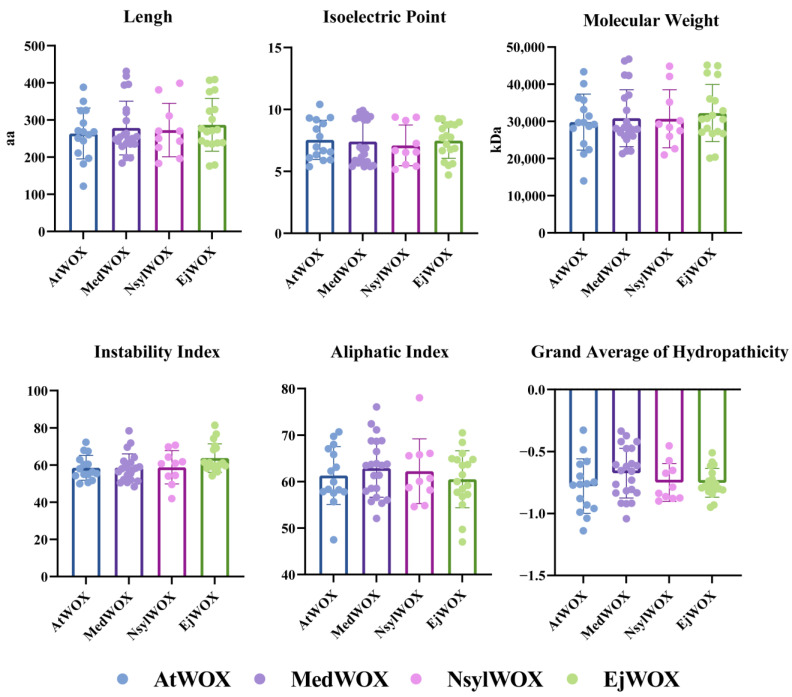
Analysis of physicochemical properties of WOX genes in *Melastoma dodecandrum*, *Arabidopsis thaliana*, *Eriobotrya japonica* and *Nicotiana sylvestris*.

Analysis of physicochemical properties of the WOX gene family of *M. dodecandrum* showed significant differences among the members (Figure 2 and Table S1). Their length ranges from 184 to 431 aa, the Molecular Weight (MW) ranges from 21,308.02 to 46,752.28 Da, and the Isoelectric Point (IP) ranges from 5.38 to 9.92. The Instability Index (II) varies from 48.38 to 78.42, the Alibaba Index (AI) fluctuates from 52.09 to 76.04 and the Grand Average of Hydrocity (GRAVY) differs from −1.042 to −0.334. Based on the species depicted in the phylogenetic tree (Figure 1), we conducted a comparative analysis of the physicochemical properties among four species. The observed trends revealed fluctuations in various protein physicochemical properties across the studied species. Notably, *M. dodecandrum* exhibited the longest protein sequence and the highest molecular weight protein within the dataset.

### 2.2. Gene Structure and Conserved Domain Analysis

**Figure 3:**
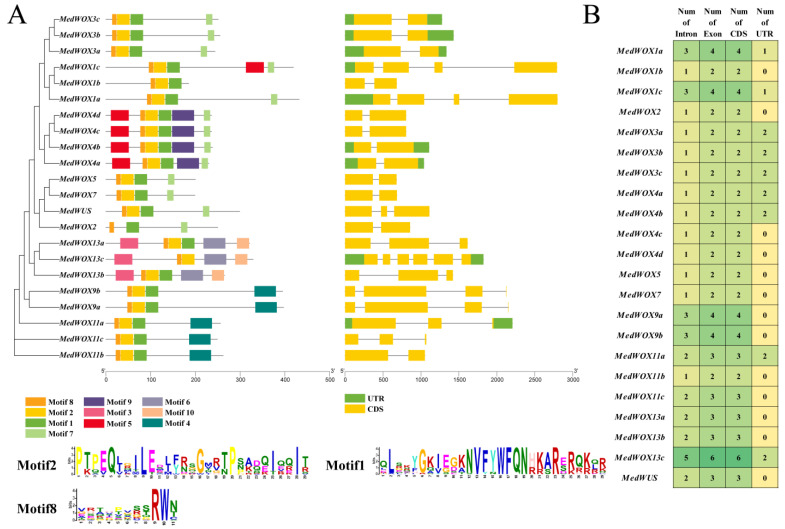
Gene structure analysis of WOX genes in *Melastoma dodecandrum*. (**A**) Phylogenetic relationships, conserved motifs and gene structure of *MedWOXs*. (**B**) Statistical analysis of intron, exon, CDS and UTR.

Based on MEME online website and TBtools software, we analyzed motif and exon-intron structures of *MedWOX* genes. The 22 WOX members contain motif8, MedWOX2 contains motif2, and MedWOX13c contains motif1 (Figure 3A). MedWOX4 (4a–4d), MedWOX13a, and MedWOX13b have the highest number of motifs, with 6. While MedWOX2 and MedWOX1b have at least 3 motifs. Notably, motifs 5, 7, and 9 were only found in the WC clade, while motifs 3, 6, and 10 were unique to the AC clade. Motif 4 was exclusive to the IC clade. One to five introns were found in all 22 *MedWOX* genes (Figure 3A). Among them, *MedWOX1c* has the longest intron. *MedWOX1a*, *MedWOX1c*, *MedWOX9a*, *MedWOX9b* and *MedWOX11a* also contain long intron, which may be due to the large number of transposable elements in these genes. Based on statistical analysis (Figure 3B), we found *MedWOX* genes contained 1–5 introns, with most *MedWOX* genes having 1–2 introns. Notably, *MedWOX13c* had the highest number of introns. Additionally, *MedWOX13c* had the greatest number of exons and CDS regions. The *MedWOX* genes had 0–2 UTRs.

**Figure 4:**
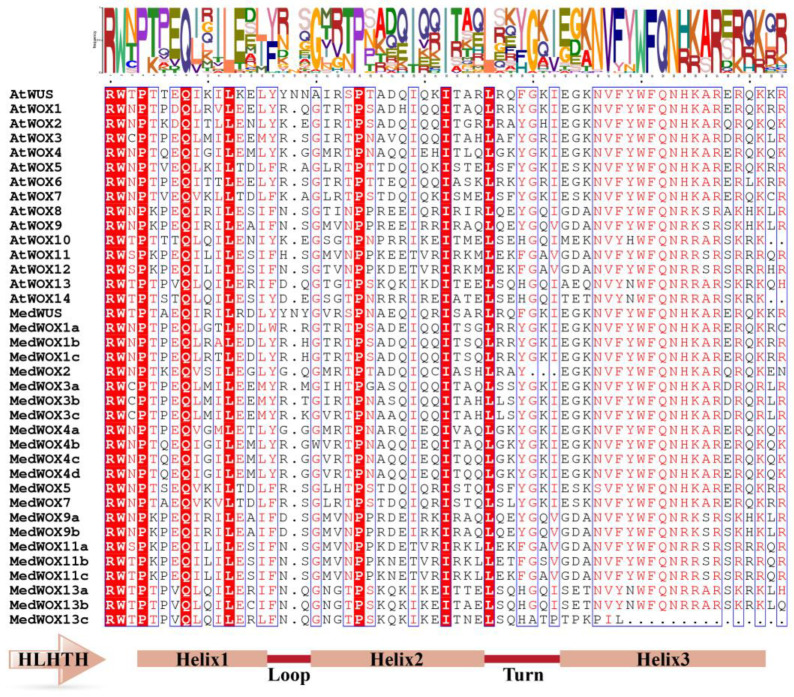
The WOX homeodomain sequence alignment analysis of *Melastoma dodecandrum* and *Arabidopsis thaliana*. The red blocks represent highly conserved residues.

The protein sequences of *M. dodecandrum* and *A. thaliana* were compared and analyzed using PhyloSuite (version 1.2.3). The results showed that the homeodomain structures of *M. dodecandrum* and *A. thaliana* were highly similar, and they both had the amino acid residue structure of Helix-Loop-Helix-Turn-Helix (HLHTH) (Figure 4). In the homeodomain structure of *M. dodecandrum*, the residues Q and L in helix 1, the residues P and I in helix 2, the residue L in the turn, and the residues W, F, Q, N and R in helix 3 exhibited a high degree of conservation. The high conservation of these residues implies their potential functional importance in *MedWOX* genes.

### 2.3. Phylogenetic Analysis of WOX Genes

The Maximum Likelihood (ML) tree was constructed using the WOX proteinss of *M. dodecandrum*, *A. thaliana*, *E. japonica* and *N. sylvestris*. The 65 WOX proteins were well clustered into three branches, including the ancient clade, intermediate clade and WUS clade [1] (Figure 1). The number of WOX proteins varies among different branches, with the largest number in the WOX4 branch, including four members (MedWOX4a, MedWOX4b, MedWOX4c and MedWOX4d). Contrarily, *A. thaliana* and *N. sylvestris* have only one member each, while *E. japonica* has two members. There was only one member in WUS and WOX2 branches of *M. dodecandrum*. Among the WOX proteins in *M. dodecandrum*, WUS clade has the most members, with 14, while ancient clade has only three members.

**Figure 5:**
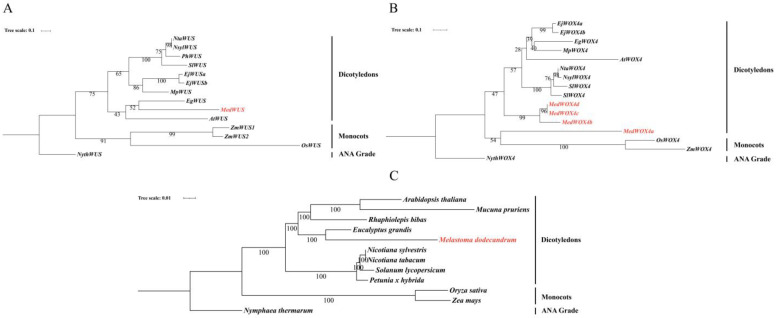
Phylogenetic Maximum Likelihood tree of single-gene and chloroplast gene for twelve species. (**A**) Phylogenetic Maximum Likelihood tree of the WUS gene for twelve species. (**B**) Phylogenetic Maximum Likelihood tree of the WOX4 gene for twelve species. (**C**) Phylogenetic Maximum Likelihood tree of the chloroplast gene for twelve species. The sources of all species protein sequences and chloroplast genomes can be found in the Table S2.

In order to further understand the evolutionary relationship between duplicated and non-duplicated WOX genes, we selected 12 species of WOX4 (duplicated in *M. dodecandrum*), WUS (non-duplicated in *M. dodecandrum*) protein sequences and chloroplast conserved genes (Table S2) to construct The Maximum Likelihood tree (Figure 5). The ML tree results of WOX4 showed that they were clustered into dicotyledons, monocots, and ANA grade branches based on species classification status (Figure 5B). There was a certain discrepancy between this result and the ML tree based on the chloroplast genes (Figure 5C). In addition, the MedWOX4 branch, containing the largest number of genes in the *M. dodecandrum* WOX family, showed evidence of expansion. The phylogenetic tree results showed that MedWOX4 was clustered to the junction of dicotyledonous WOX4s and monocotyledonous WOX4s. MedWOX4b, MedWOX4c, and MedWOX4d clustered to dicotyledonous WOX4s. But *MedWOX4a* was clustered together with monocotyledonous WOX4s. The *EgWOX4* of *Eucalyptus grandis* Hill., which is closely related to *M. dodecandrum*, was not clustered with *MedWOX4*. In the chloroplast ML tree, *M. dodecandrum* was not clustered together with monocotyledonous plants, but clustered together with *E. grandis*.

*WUS* gene has only one member (*MedWUS*) in the WOX gene family of *M. dodecandrum*, and there was no member expansion. According to the results of the phylogenetic tree, the clustering of ML tree (Figure 5A) of the WUS genes in 12 species divided into three major categories (dicotyledons, monocots, and ANA grade). In *M. dodecandrum*, MedWOX4 and MedWUS belong to the same WUS clade. But unlike MedWOX4, MedWUS and EgWUS were clustered into one branch, which was similar to the classification status of the two species. The clustering results of MedWUS and EgWUS were similar to the chloroplast ML tree.

### 2.4. Synteny Analysis

**Figure 6:**
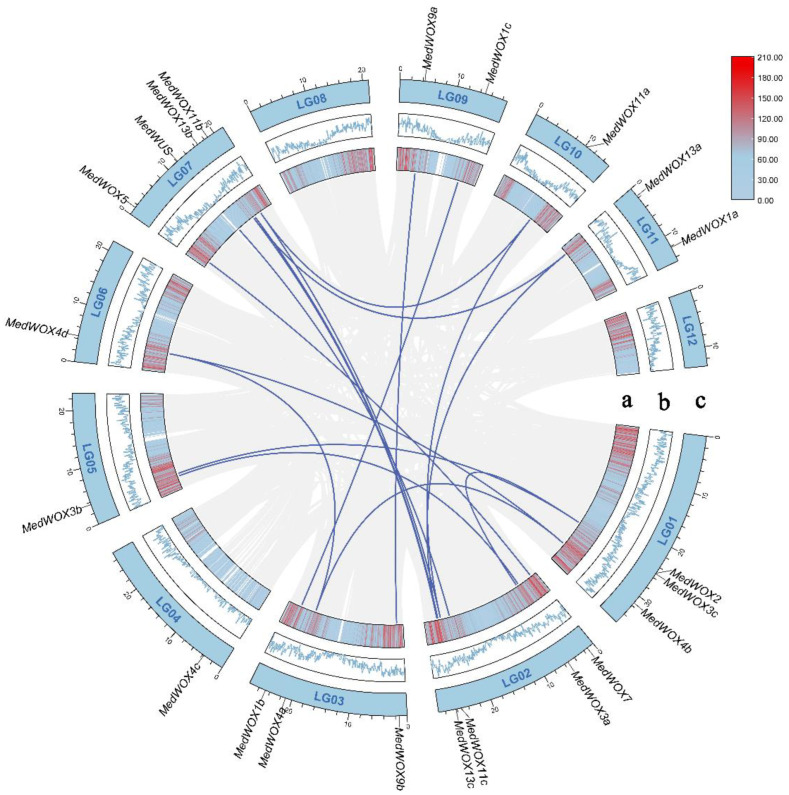
Synteny analysis of the WOX gene in *Melastoma dodecandrum*. Syntenic gene pairs are connected by blue line. a: Red line colors indicate gene, b: The blue line represents gene density. c: LG01–LG12 representative *Melastoma dodecandrum* 12 chromosomes.

The numbers of *MedWOX* genes vary greatly among different chromosomes. There are four *MedWOX* genes on chromosomes 2 and 7, three *MedWOX* genes on chromosomes 1 and 3, two *MedWOX* genes on chromosomes 9 and 11, and only one *MedWOX* gene on each of chromosomes 4, 5, 6 and 10, respectively. The rest of the chromosomes do not have the *MedWOX* gene (Figure 6). In addition, we found 15 segmental duplications on the twelve chromosomes of *M. dodecandrum*, and no gene tandem duplication was observed on the same chromosomes. Segmental duplications were identified for all *MedWOX* genes, except for *MedWOX4c* and *MedWOX1a*.

### 2.5. Cis-Acting Element Prediction of WOX Gene Family

**Figure 7:**
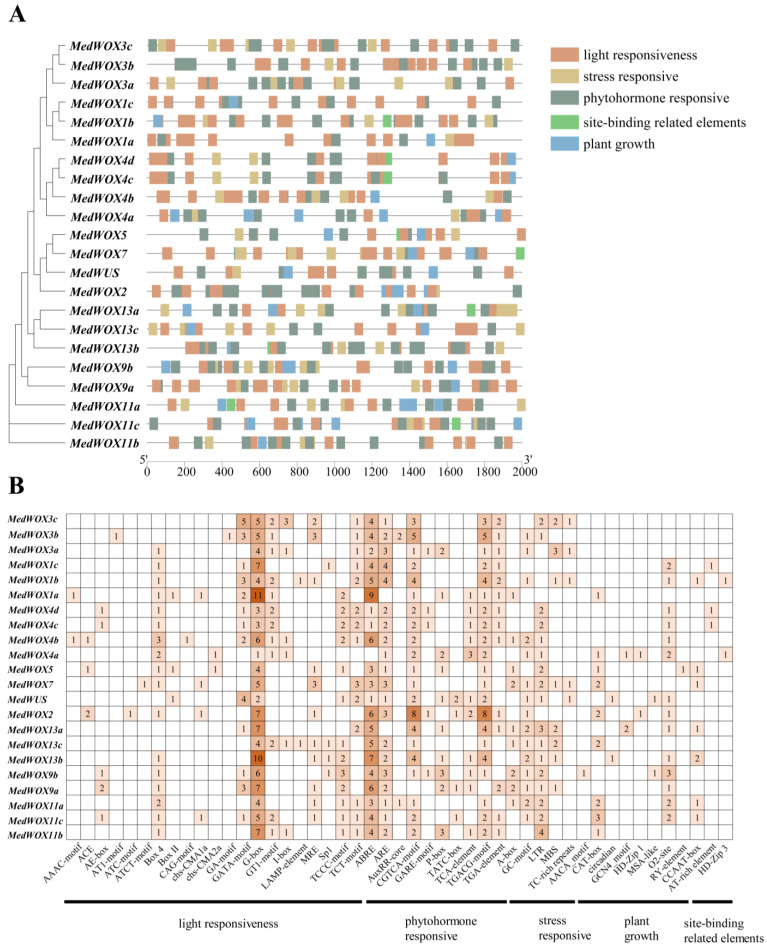
The result of cis-acting elements in promoter of the *MedWOX* genes. (**A**) Red, yellow, dark green, light green, blue bars represent the light responsive, stress responsive, phytohormone responsive, site-binding and plant growth elements in *MedWOX* promoter regions, respectively. (**B**) Number of promoter elements in *Melastoma dodecandrum*. The degree of orange color represents the number of cis-elements upstream of the *MedWOXs*.

The promoter elements were predicted in the region of 2000 base pairs upstream of the *MedWOX* gene. Our analysis identified 712 cis-regulatory elements in *M. dodecandrum* belonging to 47 different types, which could be classified into 5 major categories. These categories included light responsive (21), stress responsive (5), phytohormone responsive (10), site-binding (3), and plant growth (8) elements (Figure 7). The G-box was the most abundant light-responsive element, representing 117 out of 282 total (41.5%). The phytohormone responsive elements, including CGTCA-motif (87/292, 29.8%), TGACG-motif (51/292, 17.5%), and ABRE (51/292, 17.5%), were relatively abundant and were associated with MeJA and abscisic acid. CAT-box (20/46, 43.5%) and O2-site (15/46, 32.6%) were the most abundant in the plant growth elements, and they were related to meristematic tissue expression and zein metabolism regulation, respectively. Among the stress responsive elements, LTR elements (30/79, 38.0%) associated with low-temperature response were the most abundant. These results suggest the *MedWOX* genes likely play key roles in light and stress response, metabolic control, and development.

### 2.6. Expression Analysis and RT-qPCR

**Figure 8:**
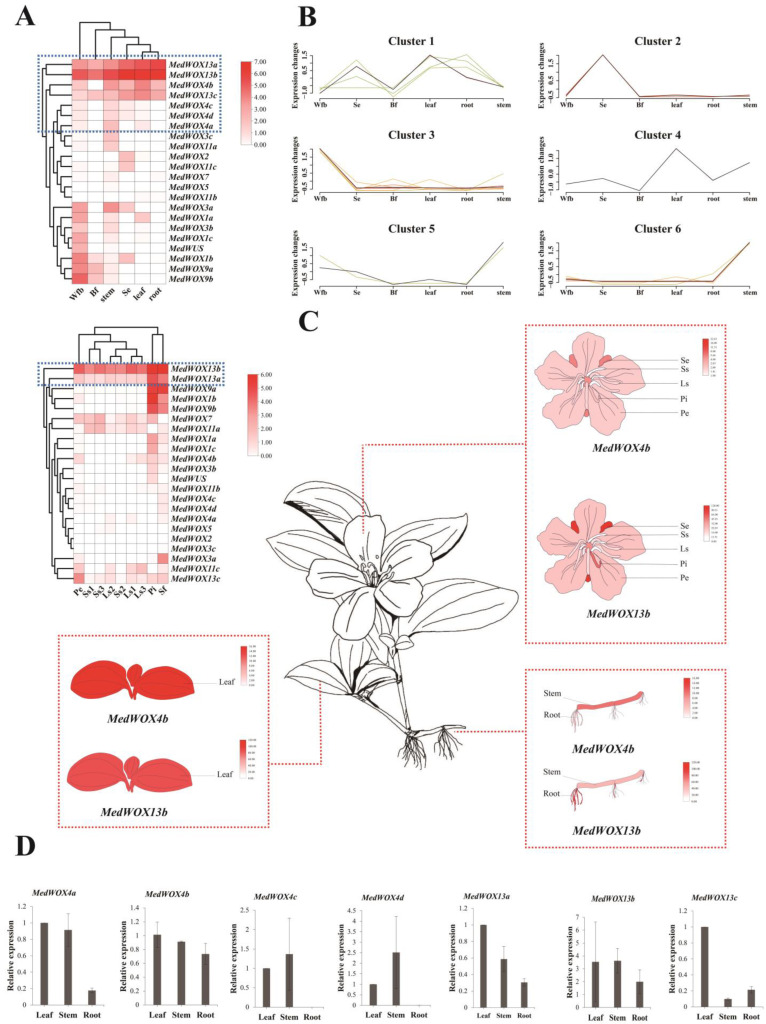
The expression patterns of *MedWOX* genes. (**A**) Expression levels of 22 *WOX* genes with indifferent plant organs and plant parts in *Melastoma dodecandrum*. We used the log2 scale, red color represents an increase in expression level, white color represents a decrease in expression level. Wfb: Whole flower bud, Ls1: Long stamen 1 (blooming), Ls2: Long stamen 2 (blooming), Ls3: Long stamen 3 (blooming), Ss1: Short stamen 1 (blooming), Ss2: Short stamen 2 (blooming), Ss3: Short stamen 3 (blooming), Pi: Pistil (blooming), Se: Sepals (blooming), Pe: Petal (blooming), Sf: Small fruit, Mf: Medium fruit (slightly colored), Bf: Big fruit (turned black). (**B**) Gene expression clustering analysis of *MedWOX* genes in indifferent plant organs and plant parts. Mfuzz of The OmicStudio tools conducted expression pattern clustering analysis(six clusters). Different lines represent different cluster results. (**C**) Expression heatmap based on transcriptome data of *MedWOX4b* and *MedWOX13b* in various plant organs of *Melastoma dodecandrum*. (**D**) Real-time fluorescence quantitative expression analysis of *MedWOXs* in different plant organs.

Based on the transcriptome data of *M. dodecandrum* (Table S3), we analyzed the different expression patterns (Figure 8A) of *WOX* genes in the roots, stems and leaves of *M. dodecandrum*. The *WOX* genes were highly expressed in the stem and entire inflorescence. Most of the genes showed low expression in long and short stamens, with some expression in pistils, sepals, petals, and fruits. Transcriptome data revealed that *MedWOX13a*, *MedWOX13b*, and *MedWOX13c* were highly expressed in flowers, fruits, leaves, roots, and stems. *MedWOX13b* showing high expression in all tested plant organs and plant parts. *MedWOX4b* was expressed in flower buds, sepals, medium-sized fruits, leaves, roots, and stems. with particularly high expression in leaves, roots, and stems. In contrast, *MedWOX4a*, *MedWOX4c*, and *MedWOX4d* were predominantly expressed in stems. *MedWOX2*, *MedWOX5*, *MedWOX11b*, and *MedWUS* showed low expression in all tissues. *MedWOX9a* and *MedWOX9b* were highly expressed in pistils and fruits. Interestingly, most of the *WOX* genes were expressed in the stem, with relatively high expression levels observed for *MedWOX3a*, *MedWOX4b*, *MedWOX13a*, and *MedWOX13b*. Gene expression clustering analysis (Figure 8B) of different plant organs and plant parts revealed that *MedWOX4b* exhibited a significant increase in expression in both leaves and stems (cluster 4). Meanwhile, *MedWOX4a*, *MedWOX4c*, and *MedWOX4d* showed a noticeable increase in expression in the stem (clusters 5 and 6). The expression levels of *MedWOX13s* were consistently moderate across most of the tissues (cluster 1). In addition, *MedWOX3a*, MedWOX3c, *MedWOX5*, *MedWOX7*, and *MedWOX11a* also exhibited a similar expression trend in clusters 5 and 6. These results have shown that *MedWOXs*, especially *MedWOX4s* and *MedWOX13s*, potential involvement in the stem growth and development of *M. dodecandrum*. Transcript levels of *MedWOX4b* and *MedWOX13b* were mapped to the model of different parts of *M. dodecandrum* (Figure 8C). We found that the expression of *MedWOX4b* was relatively high in sepals, roots, stems and leaves. However, *MedWOX13b* was highly expressed in all mapped sites, especially in sepals, roots, stems and leaves.

To validate the transcriptome data, we performed RT-qPCR experiments for *MedWOX4a*, *MedWOX4b*, *MedWOX4c*, *MedWOX4d*, *MedWOX13a*, *MedWOX13b*, and *MedWOX13c*. All seven *MedWOX* genes analyzed exhibited expression in stem tissues (Figure 8D). *MedWOX4b*, *MedWOX13a*, and *MedWOX13b* exhibited high expression in the leaves, roots, and stems, consistent with the transcriptome data. *MedWOX4a* and *MedWOX13c* showed a similar expression pattern to the transcriptome data. *MedWOX4c* and *MedWOX4d* were expressed in the leaves and stems, while the transcriptome data indicated that they were only expressed in the stems.

## 3. Discussion

The homeodomain is a typical structural domain of the WOX TFs [1], which are widely present in higher plants and play important roles in plant growth and development. In three cotton species, *Gossypium arboreum* Linn., *G. raimondii* Ulbr., and *G. hirsutum* L., 26, 31, and 50 (NAU)/33 (BJI) *WOX* genes were identified, respectively [42]. In *Brassica rapa* L. and *B. oleracea* L., 25 and 29, *WOX* genes were identified, respectively [43]. *B. napus* L. contains 58 *WOX* genes, which are classified into three main branches [44]. There were 15 *WOX* genes in *A. thaliana*. Only 11 *WOX* genes are identified in *Citrus sinensis* Pers. [45]. In this study, we identified 22 *WOX* genes with homeodomains from the entire genome of *M. dodecandrum* and divided them into three branches (ancient clade, intermediate clade, and WUS clade) (Figure 1). It can be seen that the number of *WOX* gene family members varies in different plants, which may be due to the preservation of *WOX* genes suitable for their own growth and development during plant evolution. The gene structure results indicate that the *WOX* gene family in *M. dodecandrum* has unique motifs, including motif8, motif2, and motif1. Different clades also have their own unique motifs. For example, the ancient clade has motif3, motif6, and motif10 that were not present in other clades. And the intermediate clade has unique motif4. This suggests that *WOX* genes in different clades may have different functions.

Gene duplication can promote the evolution of plant gene function, but it can also drive the emergence of new protein oligomeric states [46,47]. Segmental duplication and tandem duplication are the main mechanisms of gene family expansion. In order to elucidate the amplification of genes, we performed synteny analysis using TBtools software (version 1.120). A total of 15 pairs of segmental duplications were identified in *MedWOX* gene family, and no tandem duplication were found. Therefore, segmental duplication may be the main reason for the expansion of *MedWOX* gene family (Figure 6).

Cis-regulatory element analysis is important for studying gene function, agronomic traits and plant metabolism [48]. Our promoter analysis uncovered that the promoter region of the *M. dodecandrum WOX* gene contains a large number of light and stress response-related elements (G-box, LTR), elements associated with MeJA and abscisic acid (CGTCA-motif, TGACG-motif, and ABRE), as well as elements related to plant growth (CAT-box and O2-site). Among these elements, G-box, CGTCA-motif, TGACG-motif, and ABRE were the most abundant (Figure 7). MeJA is an important hormone for plants to respond to biotic and abiotic stress, while abscisic acid plays a crucial role in response to salt and drought stress, regulating seed dormancy and plant growth and development [49,50,51]. These results suggest the *MedWOX* genes likely play roles in the response to the light, stress and plant growth of *M. dodecandrum*.

*WOX* genes play a crucial role in regulating stem cell growth and development, controlling leaf growth, and promoting root formation [52,53,54]. *WOX4* gene is a member of the WUS clade. It has been reported to be expressed in the vasculature of roots and stems in *A. thaliana* and *Solanum lycopersicum* L., and may promote differentiation and maintenance of the vascular procambium [15]. In Populus trees(*Populus tremula* L. × *P. tremuloides* Michx and *P. alba* L. × *P. glandulosa* Moench clone 84K), ubiquitinated *PagDA1* can antagonize *PagWOX4* in a common pathway to regulate cambial activity. *PttWOX4* gene can control cell division activity in the vascular cambium, thus promoting stem growth [55,56]. However, only one or two *WOX4* genes have been identified in many species, such as *A. thaliana*, Paper Mulberry(*Broussonetia kazinoki* Siebold × *B. papyrifera* (L.) Vent.) [57], and Bambusoideae [58]. In this study, Our phylogenetic analysis categorized the 22 identified *MedWOX* genes into three branches and nine types. Among them, the *MedWOX4* type has four members (*MedWOX4a*, *MedWOX4b*, *MedWOX4c*, *MedWOX4d*) (Figure 1). This suggests that *WOX4* gene have undergone significant expansion in *M. dodecandrum*, which may be related to the whole-genome duplication events that *M. dodecandrum* experienced [59]. Transcriptome data and RT-qPCR results showed that *MedWOX4a*, *MedWOX4b*, *MedWOX4c* and *MedWOX4d* were all expressed in different parts of *M. dodecandrum*, while the expression of the four genes was relatively high in the stem (Figure 8). We speculate that the amplification of *MedWOX4* may be related to the growth of the stem of *M. dodecandrum*.

The ancient clade was typically found in lower plants, while members of the WUS clade and intermediate clade have differentiated from members of the ancient clade that underwent expansion during evolution [60]. Plant evolution may also promote gene evolution. The results of the single-gene phylogenetic tree showed that the *WOX4* and *WUS* genes cluster well according to ANA-grade, dicotyledons and monocots(Figure 5A,B). *WOX4* underwent duplicattion during the evolutionary process of *M. dodecandrum*. But the duplicated *MedWOX4* and the non-duplicated *MedWUS* were found to occupy similar positions in the phylogenetic tree. This indicates that when a species evolved to a certain extent, it may have driven the evolution of the *WOX* gene. This allows the *WOX* gene to reflect a certain position in species evolution. However, whether the gene is replicated or not has little impact on the position of *WOX* in the phylogenetic tree. Constructing a phylogenetic tree based on chloroplast genes to illustrate the evolutionary position of species is a highly credible approach. The results of the single-gene phylogenetic tree have a slight difference when compared to the results obtained from the chloroplast gene phylogenetic tree (Figure 5C). The results of the single-gene phylogenetic tree has a little difference when compared to the results obtained from the chloroplast gene phylogenetic tree (Figure 5C). The discordance between the single-gene ML tree and chloroplast gene ML tree highlights the limitations of using phylogenies of individual genes to analyze gene evolution.

Previous studies found that *WOX13* regulated plant stem cell growth and promoted plant callus, organ growth and development [33,34]. *GhWOX13a* and *GhWOX13b* showed higher expression in the roots and stems, with specific expression in cotton fibers (*G. hirsutum*) [61]. *PgWOX13a* and *PgWOX13b* were detected in the thin-walled cells of the main root and cultured adventitious roots of *Panax ginseng* C.A.Mey. seedlings, indicating their important role in maintaining the differentiation and self-renewal of the cortex and xylem [62]. A recent study showed that *WOX13* could regulate *WUS* and negatively regulated the expression of regulators in shoot meristem, thus affecting shoot regeneration [16]. Those findings of these studies suggest that *WOX13* plays a crucial role in regulating the growth of plant stems and buds. Therefore, elucidating the precise mechanisms underlying the function of *WOX13* could provide valuable insights into the adaptive strategies employed by pioneer species to thrive in novel habitats. Based on transcriptomic data and RT-qPCR results, we found that *MedWOX13a*, *MedWOX13b*, and *MedWOX13c* were highly expressed in flowers, fruits, leaves, roots, and stems (Figure 8), similar to previous studies. Those are the indispensable important organs in plants. It showed that the *MedWOX13s* play an important role in growth and development of *M. dodecandrum*. It should be noted that *MedWOX13s* was similar to *MedWOX4s* in that they had higher expression in the stems. This suggested the *MedWOX13s* and *MedWOX4s* may have played a role in promoting stem growth. This may help the adventitious roots on the stems of M. dodecandrum to better set on land, thereby promoting the rapid spread and prostrate establishment of *M. dodecandrum* in new habitats However, further investigations are required to substantiate this hypothesis.

## 4. Materials and Methods

### 4.1. Identification and Classification of WOX Genes in M. dodecandrum

The *M. dodecandrum* genome data used in this study were from our own research group and were published in 2022 by Hao et al. [59]. The *M. dodecandrum WOX* genes were identified through the BLASTP and Simple HMM Search function of TBtools (version 1.120) [63]. *A. thaliana* WOX protein sequences were retrieved from The Arabidopsis Information Resource (TAIR; https://www.arabidopsis.org/, accessed on 8 April 2023). *A. thaliana* WOX protein sequences were used as query sequences to perform the BLASTp search (E-value < 1 × 10^−5^, Num of Hits: 500, Num of Aligns: 250) with all protein sequences of *M. dodecandrum*. Performed an HMM Search on the entire protein sequence of *M. dodecandrum* using the Hidden Markov Model (PF00046), with default settings. Each AtWOX protein was used as a query to BLASTp search against the *M. dodecandrum* genome. For each AtWOX, 19–25 matches were obtained, many of which were the same ID. We removed the same BLASTp results and abtained unique BLASTp results. HMM Search results confirmed that those unique BLASTp results matched the Hidden Markov Model. Then, we obtained the unique results containing gene ID information. Protein sequences of these IDs were extracted using the Fasta Extract tool of TBtools (version 1.120). The protein sequences were checked for the presence of amino acid residue structure of Helix-Loop-Helix-Turn-Helix, which was typical domain of WOX transcription factors (homeobox domain), by sequence alignment analysis of PhyloSuite (version 1.2.3) [64], with default settings. Then, we removed protein sequences without the HLHTH structure, and finally obtained the *M. dodecandrum WOX* genes (Table S5). *MedWOX* genes were named and classified based on the conventions used for *A. thaliana WOX* genes, Cao et al., Li et al. and Wang et al. [37,44,58].

### 4.2. Gene Structure and Conserved Domain Analysis of MedWOX Genes

The exon-intron structures and physicochemical properties analyses of WOX proteins were performed using TBtools (version 1.120). Physicochemical properties analyses was visualized by GraphPad Prism 9.0.0. The NCBI Conserved Domain Database CDD (https://www.ncbi.nlm.nih.gov/cdd, accessed on 22 June 2023) was used to predict the conserved domains of WOX genes. Multiple Em for Motif Elicitation (MEME, https://meme-suite.org/meme/tools/meme, accessed on 23 June 2023) was used to analyze the conserved motifs of the WOX genes. Multiple sequence alignments were generated with PhyloSuite (version 1.2.3) and visualized by ESPript 3.0 [65], and the sequence logo was visualized by the LogoJS website (https://logojs.wenglab.org/, accessed on 15 July 2023).

### 4.3. Phylogenetic Analysis

*E. japonica* and *N. sylvestris* WOX protein sequences were downloaded from the resources provided by Yu et al. [66] and Li et al. [67]. A phylogenetic tree was inferred using PhyloSuite (version 1.2.3, model: JTT+F+R5, bootstrap was set to 1000) with protein sequences from *M. dodecandrum*, *A. thaliana*, *E. japonica* and *N. sylvestris*. We selected WOX4, WUS protein sequences and chloroplast genomes from a total of twelve species (Table S2) including ANA Grade, monocots, and dicotyledons to construct The Maximum Likelihood (ML) tree by PhyloSuite(version 1.2.3). All of the phylogenetic trees were visualized using the iTOL website (https://itol.embl.de/, accessed on 1 June 2023).

### 4.4. Synteny Analysis of MedWOX Genes

Obtained the positional information of *MedWOX* based on the genome annotation file. Generated synteny files and gene pair files using the Run MCScanx Wrapper function in TBtools (version 1.120). The synteny relationship between the genes were visualized by TBtools (version 1.120).

### 4.5. Cis-Acting Element Prediction of MedWOX Genes

The 2000 bp upstream sequence of WOX genes in *M. dodecandrum* was obtained by TBtools (version 1.120). Putative cis-regulatory elements in the *MedWOX* genes promoters were identified using PlantCARE (http://bioinformatics.psb.ugent.be/webtools/plantcare/html/, accessed on 14 July 2023). Data analysis and visualization were conducted in TBtools (version 1.120) and Excel 2021.

### 4.6. Expression Analysis and RT-qPCR

The transcription data of *M. dodecandrum* (Table S3) were from our own research group and were published in 2022 by Hao et al. [59]. Expression Analysis for the 22 *MedWOX* genes in different plant organs and plant parts of *M. dodecandrum* were analyzed and visualized by TBtools (version 1.120). were The OmicStudio tools (https://www.omicstudio.cn/tool, accessed on 10 October 2023) was used for Mfuzz analysis. The plant materials used in this study were obtained from the National Orchid Germplasm Resources of Fujian Agriculture and Forestry University, Fuzhou, China. Seven *MedWOX* genes (*MedWOX4a*, *MedWOX4b*, *MedWOX4c*, *MedWOX4d*, *MedWOX13a*, *MedWOX13b*, and *MedWOX13c*) were chosen for RT-qPCR validation. Total RNA was extracted using the OMEGA R6827 Plant RNA Kit, following the second method described in the kit manual for difficult samples. cDNA synthesis was performed using the TB Green qPCR method, and RT-qPCR analysis was conducted using the Hieff UNICON® Universal Blue qPCR SYBR Green Master Mix. All experiments were performed with three biological replicates. The Ct values obtained from RT-qPCR were processed and analyzed (using the formula 2^−ΔΔCT^) to determine the relative expression levels of the seven genes in different tissues. Primers for RT-qPCR were designed using Primer3Plus (https://www.primer3plus.com/, accessed on 29 June 2023) and internal reference gene were followed Hao et al. [59]. All RT-qPCR primers information were in Table S4.

## 5. Conclusions

Based on the genomic data of *M. dodecandrum*, we identified the *WOX* gene family of *M. dodecandrum*. Our analysis encompassed the physicochemical properties, conserved domains, gene structure, sequence and synteny analysis and so forth of the *WOX* genes in *M. dodecandrum*. We constructed phylogenetic trees of *M. dodecandrum* with *A. thaliana*, *E. japonica*, and *N. sylvestris*, as well as single-gene phylogenetic trees of *WOX4* and *WOX13* in twelve plants. Notably, we observed substantial expansion of the WOX4 clade in *M. dodecandrum*. Promoter element prediction indicated that the *WOX* genes of *M. dodecandrum* may be associated with light and stress response and plant growth. Based on transcriptomic and RT-qPCR data, The *WOX* genes, especially *MedWOX4* and *MedWOX13*, had high expression in the stems of *M. dodecandrum* and *MedWOX4* expressed specifically in the stem. This suggested that these genes may be related to stem growth. The growth of the stems could have helped the adventitious roots of the *M. dodecandrum* to anchor better, contributing to its creeping growth habit. These findings provide potential research directions for unraveling the role of WOX transcription factors in plant growth and development.

## Data Availability

The original genome sequences described in this article have been abtained from National Genomics Data Center (NGDC, https://ngdc.cncb.ac.cn, accessed on 8 April 2023) under accession number PRJCA005299, CRA004277, CRA004347 (including whole genome and assembly data). All data generated or analyzed during this study are included in this published article (Supplementary Files) and also available from the corresponding author on reasonable request.

## Supplementary Materials

The following supporting information can be downloaded at: https://www.mdpi.com/article/10.3390/ijms242417349/s1.
